# 

**Published:** 1872-01

**Authors:** 


### INDEX TO VOLUME XIX.

| | PAGE |
|---|---|
| Amusement | 46 |
| Accuracy | 62 |
| Alcohol | 121 |
| Autumnal Wisdom | 261 |
| Agriculture | 271 |
| Bathing | 80 |
| Beef Tea | 84 |
| Biliousness | 97 |
| Bed Bugs | 140 |
| Bruises | 204 |
| Brain-worker | 213 |
| Bread | 236 |
| Buried Alive | 239 |
| Bright's Disease | 276 |
| Business Thrift | 277 |
| Chapped Hands | 20 |
| Communism | 28 |
| Cold Feet | 36 |
| Catarrh | 64 |
| Changing Clothing | 65 |
| Consumption | 73, 284 |
| Constipation | 78 |
| Cancer | 86, 107 |
| Colds | 89, 238 |
| Cholera | 179 |
| Contagious Diseases | 222 |
| Church-goers | 225 |
| Churches, Heated | 246 |
| Cold Water | 248 |
| Cripples | 282 |
| Drunkenness | 114, 131, 155 |
| Death's Hour | 131 |
| Drowning | 182 |
| Diabetes | 186 |
| Diarrhœa | 196 |
| Dressing Warm | 275 |
| Dress-making | 278 |
| Emergencies | 14 |
| Eggs, Preserved | 22 |
| Eight Hour Law | 27 |
| Education, The Best | 140, 151 |
| Eating | 247 |
| Education and Health | 266 |
| Fault-Finding | 19, 204 |
| Farewell to Books | 25 |

| | PAGE |
|---|---|
| Feet, Cold | 36 |
| Fanatics | 61 |
| Fat People | 110 |
| Flowers a Medicine | 110 |
| Filth Diseases | 112 |
| Fruits Healthful | 137 |
| Fever Cure | 186 |
| Fevers | 195 |
| Fever and Ague | 199 |
| Family Trouble | 218 |
| Florence Nightingale | 288 |
| Good Humor | 178 |
| Good Nursing | 169 |
| Healthful Houses | 40 |
| Hay Fever | 44 |
| Homeless | 88 |
| Health Teachers, Blind | 90 |
| Hereditary Influences | 153 |
| House Poisons | 189 |
| Hair Dyes | 191, 283 |
| Hurrying | 200 |
| Handsome Face | 216 |
| Home Training | 259 |
| Investing Money | 3, 86 |
| Insane Women | 33 |
| Incurable | 38 |
| Immortal Life | 45 |
| Indoor Amusement | 46 |
| Itch | 230 |
| Joking | 106 |
| Living Together | 17 |
| Learn a Trade | 22 |
| Life's Tenure | 47 |
| Life's Work | 82 |
| Lead Pipes | 96 |
| Lungs | 105 |
| Lock-Jaw | 206 |
| Louse | 226 |
| Model Wife | 48 |
| Marriage Subjects | 91, 103 |
| Mercury | 140 |
| Microscope | 158 |
| Mysterious Influences | 202 |
| Movement Cure | 213 |

PAGE
Mothers .......................... 272
Mad Dogs .......................... 274

Neatness .......................... 68
Nursing .......................... 169, 288

Oneness of Disease .......................... 193

Paste .......................... 9
Pure Port Wine .......................... 59
Physicians, Educated .......................... 67
Pneumonia .......................... 133
Pews in Church .......................... 215
Parasites .......................... 226
Paralysis .......................... 250
Potatoes .......................... 268

Ringworm .......................... 233
Ruptures .......................... 282

Scrap-Book .......................... 12
Sick-Chambers .......................... 21, 169
Small-Pox .......................... 55
Scarlet Fever .......................... 57
Sunny Face .......................... 66
Sleeplessness .......................... 79
Squeaking Shoes .......................... 82
Success in Business .......................... 84
Soft Palate .......................... 93
Sleep .......................... 140

PAGE
September, Baleful .......................... 209
Sick Headache .......................... 240
Sextant Literature .......................... 241
Sleeping-Rooms .......................... 273

Typhoid Fever .......................... 49
The Good Waldensian .......................... 57
Tooth-ache .......................... 85
Trichina .......................... 87
Taking Cold .......................... 89
Turkish Baths .......................... 113
Training Girls .......................... 270

Unfortunate Persons .......................... 32
Ulcers .......................... 258

Venerable Men .......................... 1
Vaccination .......................... 132
Ventilation .......................... 241

Wives' Worries .......................... 7
Walking .......................... 77
Warming Houses .......................... 134
Wedding Day .......................... 145
Warts .......................... 166
Worms .......................... 187
Warning Knock .......................... 212
Watering Places .......................... 223
Wheat and Bread .......................... 236
Wounds .......................... 258

ERRATUM.—On page 164, in the July number, substitute "Oxygen as an Anæsthetic," in the place of "Ether."

below the surface. The keg should be kept in a cool place in the cellar. The liquor will not freeze except at a lower temperature than freezing point.

---

### BOOK NOTICES.

"The Right One;" by MARIE SOPHIE SCHWARTZ. $1.50, 8vo. Lee & Shepard, Boston and New York. Of all novels those of this beautiful writer are the least injurious, the purest, and the best; and this volume is one of the few which can be safely read by the young.

J. S. Redfield, 140 Fulton Street, has published "Christine," from the French of Louis Enault, pronounced a "charming story." Mr. Redfield has also published the "Traveller's Guide to the City of New York." 25 cents. An accurate, succinct, and yet full description of all that is interesting in the metropolis of the nation. A more reliable and satisfactory guide for all who visit New York for the first time has not yet been published. Persons who have already visited New York could not do better than to order this timely publication, as its reading will help to fix in the mind what has been seen, and will enable the visitor to repeat the visit, in imagination, at his own fireside.

"Health and Comfort at Home," with suggestions and selections from various authors, a ten-cent tract of 27 pages, by ROSS WINANS, of Baltimore, Maryland, is well worthy to be read and studied by all who are in search of a home well constructed and well ventilated.

Miller, Haynes & Co. have published the following tracts, six in number, which will be sent postpaid, for one dollar: —

"A Mother's Advice for every Girl." "A Father's Advice for every Boy." "Vital Force, how Wasted, how Preserved." "How to Bathe;" being a family guide to the use of water in preserving health and treating disease. "Dyspepsia;" its varieties, causes, symptoms, and treatment, by Hydropathy and Hygiene. "Turkish Bath;" how to take it, when and where. These are useful and safe, and can be read with profit by all.

"Sam Slick of Slickville, his Sayings and Doings;" with illustrations, by Darley. Hurd & Houghton, publishers, New York, have done the reading public a favor, in republishing a book which found many thousands of admiring readers a quarter of a century ago. It is the most instructive, the most mirth-provoking, and health-promoting book of the times.

"Presbyterian Historical Almanac for 1872;" by JOSEPH M. WILSON, Philadelphia, Pennsylvania. This is an annual which merits the patronage of every intelligent Presbyterian. For his industry, and the labor he has expended on these annuals, Mr. Wilson deserves the thanks and the patronage of the whole Church, especially as heretofore they have been published at a pecuniary loss to him. Every clergyman and every elder in the Church ought to have the whole set of these valuable, historical, and statistical annuals.

"Health without Medicine, or How to Treat the Sick without Medicine," is a handsome volume of 537 pages, by Dr. JAMES C. JACKSON, of Dansville, N. Y., where is located the most successful water-cure establishment in our country. This book indicates the treatment of disease by the judicious use of water and vegetarian diet; and to persons who favor Hydropathy, it will be of great value, because it treats plainly of the symptoms and nature of our diseases, and how by the judicious employment of water, of air, exercise, and diet, the sick may get well. It is valuable also for its strong testimony against the use of tobacco and ardent spirits, in any and all forms.

The following dialogues, just published, written by Mrs. NELLIE H. BRADLEY, author of "The First Glass," "Young Teetotaler," "Reclaimed," "Marry no Man if he Drinks," etc., will be found equal to her previous writings: "Wine as a Medicine; or, Abbie's Experience." 10 cents; $1.00 per dozen. "The Stumbling-Block; or, Why a Deacon Gave up his Wine." 10 cents; $1.00 per dozen. The following are among the best dialogues for Lodges, Divisions, and Temperance Societies: "The First Glass; or, The Power of Woman's Influence." "The Young Teetotaler; or, Saved at Last." 15 cents for both; $1.50 per dozen. "Reclaimed; or, The Danger of Moderate Drinking." 10 cents; $1.00 per dozen. "Marry no Man if he Drinks; or, Laura's Plan, and how it Succeeded." 10 cents; $1.00 per dozen. "Which Will You Choose?" 36 pp. By Miss DWINELL MARY CHELLIS. 15 cents; $1.50 per dozen. "Trial and Condemnation of Judas Woemaker." 15 cents; $1.50 per dozen. Also "National Temperance Almanac" for 1872. 10 cents. The above will be sent by mail, post-paid, to any address, on receipt of price. Address J. N. Stearns, 58 Reade Street, New York.

"A Brief History of the United States;" 350 pages, sent by mail, for $1.50. Address A. S. Barnes & Co., New York and Chicago. Designed for schools and families; it is one of the best books of its class, and brings down the history of our country to the present time of General Grant's administration.

SWINTON'S "Condensed History of the United States," by Ivison, Blakeman, & Taylor, 140 Grand Street, New York; designed also for schools. It is succinct, accurate, and full, and will be of great value to all as a book of reference, and no doubt will be a standard volume for many years to come.

"Walks in Rome;" 678 pages, 12mo. AUGUSTUS J. C. HARE, author, published by George Routledge & Sons, 416 Broome Street, New York, is one of the most instructive and deeply interesting books of travel lately issued from the American Press. No one who has ever been to Rome, no one who hopes to get there, no intelligent traveller now there, can afford to be without this valuable handbook of the most noted city in the world's history. The details are so full, so accurate, and so comprehensive, that any one taking the volume and making it his guide, will not only not miss seeing everything worth seeing, but will have seen it in such a manner, so pleasantly connected with historical incidents belonging to each particular locality, that the impressions will not be erased in a lifetime; to be talked of and thought about in all after years, with a pleasure and an intelligence not possible without reading Mr. Hare's "Walks in Rome," all the more agreeable from its being affectionately dedicated to his "Mother."

